# Supplementary material for: Broad Spectral Response FeOOH/BiO2−x Photocatalyst with Efficient Charge Transfer for Enhanced Photo-Fenton Synergistic Catalytic Activity
Source: Molecules. 2024 Feb 19;29(4):919. doi: 10.3390/molecules29040919 (PMC10893118; doi:10.3390/molecules29040919)
Supplement: Supplementary file 1 [file molecules-29-00919-s001.zip › molecules-2840723-supplementary.pdf]

Supplementary material

for

Broad spectral response FeOOH/BiO_{2-x} photocatalyst with efficient charge transfer for enhanced photo-Fenton synergistic catalytic activity

Pengfei Wu[1,2], Yufei Qin[2], Mengyuan Gao[3], Rui Zheng[2], Yixin Zhang[2], Xinli Li[2], Zhaolong Liu[2,4], Yingkun Zhang[4], Zhen Cao[2,*], Qingling Liu[1,*]

[1]. Tianjin Key Lab of Indoor Air Environmental Quality Control, School of Environmental Science and Engineering, Tianjin University, Tianjin 300350, China;

[2]. Hebei Pollution Control Technology Innovation Center of Steel and Coking Industry, Department of Environmental and Chemical Engineering, Hebei Vocational University of Industry and Technology, Shijiazhuang 050091, China;

[3]. Hebei Provincial Academy of Ecological Environmental Science, Shijiazhuang 050030, China;

[4]. Hebei Key Lab of Environmental Photocatalytic and Electrocatalytic Materials, College of Chemical Engineering, North China University of Science and Technology, Tangshan 063210, China.

[*]. Correspondence:
liuql@tju.edu.cn; Tel: +86-22-8740 1255；
caozhen81112@126.com; Tel: +86-311-8527 6051.

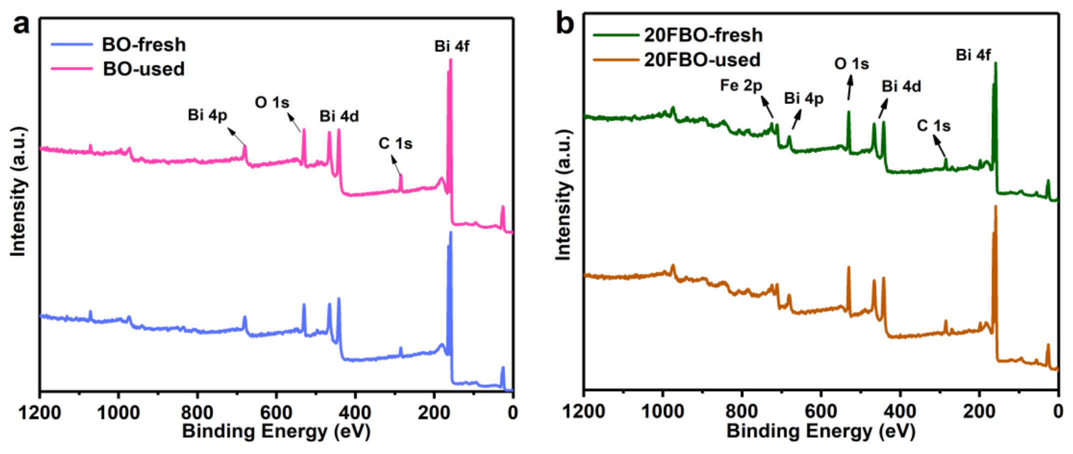

Figure S1. Full XPS spectrum of (a) BO and (b) 20FBO.

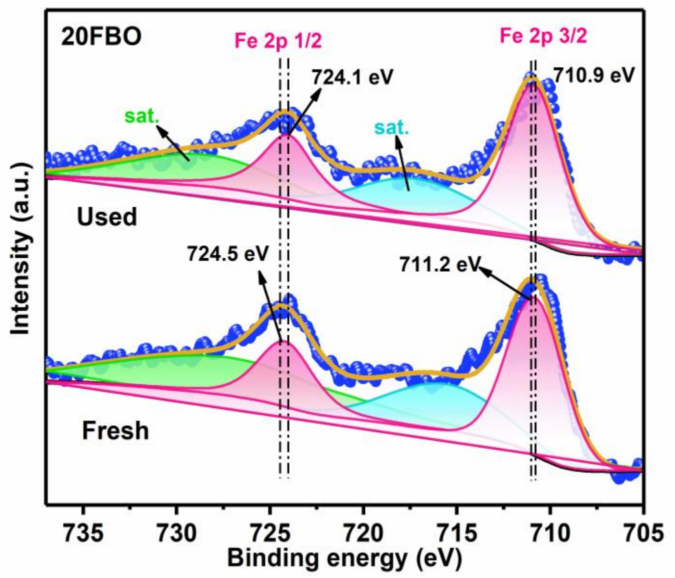

Figure S2. High-resolution XPS spectra of Fe 2p over 20FBO.

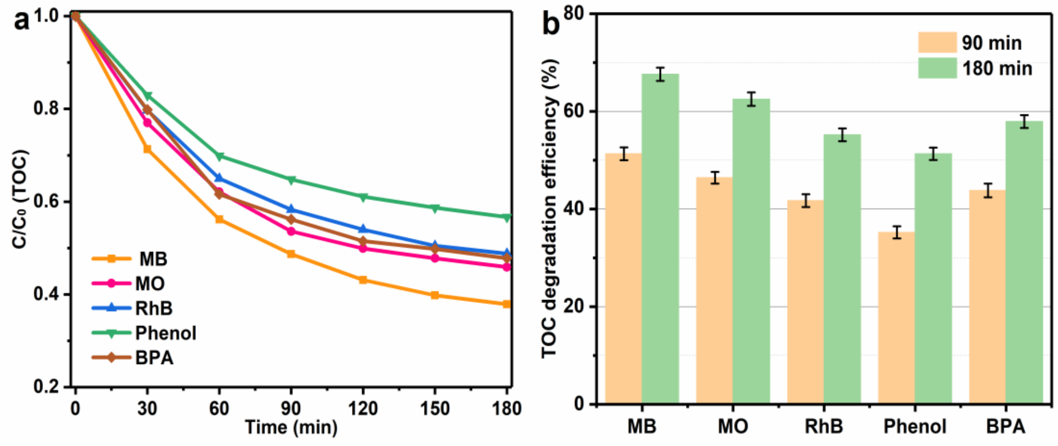

Figure S3 (a) Time profiles of TOC removal to different contaminants under photo-Fenton system; (b) Comparing the TOC removal efficiency of photo-Fenton system to different contaminants. (The yellow

bar graph represents the TOC removal rate of the system when the H2O2 concentration is 8mM for 90 minutes. The green bar graph represents the removal rate of the same volume of H2O2 added again after 90 minutes of reaction)

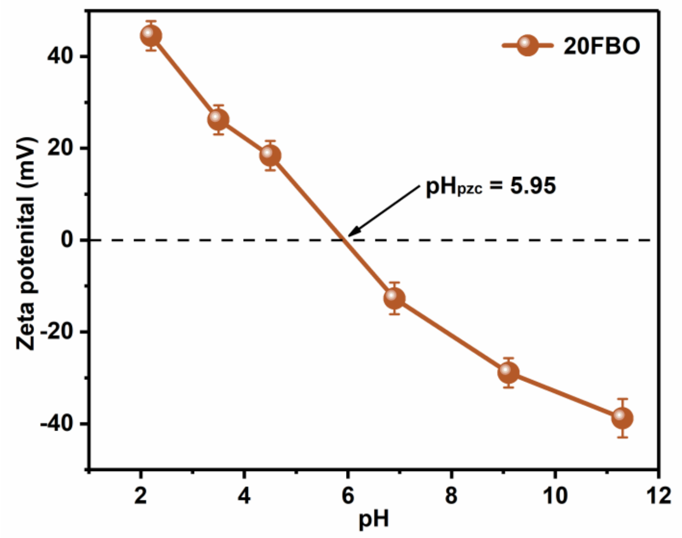

Figure S4. Zeta potentials of 20FBO suspensions with different pH values.

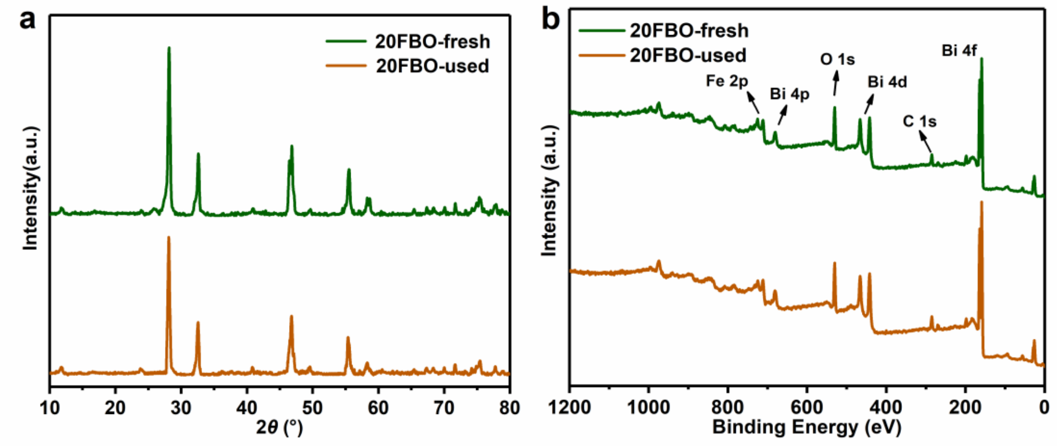

Figure S5. (a) XRD spectrum; (b) XPS spectrum of 20FBO before and after MB degradation.

Table S1 Comparison of degradation performance of MB using different photocatalysts

Catalysts	Reaction system				reaction time (min)	Degradation rate (%)	reference
	Catalyst dosage (mg)	H_2O_2 (mmol/L)	MB concentration (mg/L)	MB volume (ml)			
TiO_2-rGO Sol−Gel	20	/	5	200	120	86	[67]

Fe_3O_4@void@CdS	10	300	10	50	20	100	[68]
RGO/Fe_3O_4	25	10	20	100	120	96	[69]
CQDs/$Bi_2O_2CO_3$	50	/	10	50	120	94	[70]
α-Fe_2O_3@GO	100	1.1	40	400	80	94	[71]
Ag@AgCl/$Bi_2O_2CO_3$	400	/	10	100	20	97	[72]

[67] S. Zhang, J. Xu, J. Hu, C. Cui, H. Liu, Interfacial Growth of TiO2-rGO Composite by Pickering Emulsion for Photocatalytic Degradation, Langmuir, 33 (2017) 5015-5024.

[68] W. Shi, D. Du, B. Shen, C. Cui, L. Lu, L. Wang, J. Zhang, Synthesis of Yolk-Shell Structured Fe3O4@void@CdS Nanoparticles: A General and Effective Structure Design for Photo-Fenton Reaction, ACS applied materials & interfaces, 8 (2016) 20831-20838.

[69] X. Jiang, L. Li, Y. Cui, F. Cui, New branch on old tree: Green-synthesized RGO/Fe3O4 composite as a photo-Fenton catalyst for rapid decomposition of methylene blue, Ceram. Int., 43 (2017) 14361-14368.

[70] Z. Zhang, S. Lin, X. Li, H. Li, T. Zhang, W. Cui, Enhanced Photocatalytic Activity toward Organic Pollutants Degradation and Mechanism Insight of Novel CQDs/Bi(2)O(2)CO(3) Composite, Nanomaterials (Basel), 8 (2018).

[71] Y. Liu, W. Jin, Y. Zhao, G. Zhang, W. Zhang, Enhanced catalytic degradation of methylene blue by α-Fe2O3/graphene oxide via heterogeneous photo-Fenton reactions, Appl. Catal., B, 206 (2017) 642-652.

[72] S. Lin, L. Liu, Y. Liang, W. Cui, Z. Zhang, Oil-in-Water Self-Assembled Synthesis of Ag@AgCl Nano-Particles on Flower-like Bi(2)O(2)CO(3) with Enhanced Visible-Light-Driven Photocatalytic Activity, Materials (Basel), 9 (2016).
